# Upregulation of long noncoding RNA Xist promotes proliferation of osteosarcoma by epigenetic silencing of P21

**DOI:** 10.18632/oncotarget.20738

**Published:** 2017-09-08

**Authors:** Tianyang Xu, Wenwei Jiang, Lin Fan, Qiuming Gao, Guodong Li

**Affiliations:** ^1^ Department of Orthopedics, Shanghai Tenth People's Hospital, Tong Ji University School of Medicine, Shanghai 200072, People's Republic of China

**Keywords:** osteosarcoma, Xist, proliferation, EZH2, P21

## Abstract

Recent studies show that lncRNAs involve in the initiation and progression of various cancers including osteosarcoma (OS). IncRNA Xist has been verified as an oncogene in several human cancers, and its abnormal expression was closely associated with tumor initiation and progression. Nevertheless, the role of Xist in OS remains unclear. Here, we revealed the Xist expression level was up-regulated in OS tissues and discovered that Xist knockdown significantly repressed OS cell proliferation. Additionally, mechanistic analysis revealed that Xist can repress P21 expression to regulate OS cell cycle and proliferation by binding to EZH2. Taking all into account, Xist may function in promoting OS cell proliferation and may potentially serve as a novel biomarker and therapeutic target for OS.

## INTRODUCTION

Osteosarcoma (OS) is the most common primary malignant bone tumor in children and young adults, about 80 percent of OS occurs in the long bones of the limbs, most often at the metaphysis of the long bones around the knee, and the other 20 percent occurs in the axial bone and pelvis [1]. Due to recent advancements in multimodal treatments, the 5-year survival rate of OS patients has significantly improved over the past decades [2]. OS is a high metastatic potential tumor, and the most common targets are the lung and other bones [3, 4]. There are a large number of OS patients experienced distant metastasis or local relapse after intensive chemotherapy and curative resection of the primary tumor [5]. The exact mechanism of the pathogenesis and progression in OS remains unclear. Therefore, there is an urgent need to explore the potential molecular mechanisms in OS and it is critical to identify new biomarkers and therapeutic targets.

Long non-coding RNA (lncRNA) is a class of RNA molecules that with more than 200 bases. They have limited or lack protein-coding capacity, but they regulate gene expression at various levels in the form of RNA (epigenetic regulation, transcriptional regulation, post-transcriptional regulation, etc.) [6]. Increasing evidence shows that lncRNAs are involved in most biological processes of cancer initiation, progression, and metastasis [7]. LncRNAs also can function as a molecular sponge for miRNAs or action as a competing endogenous RNA to regulate its target downstream molecules expression [8, 9]. Indeed, accumulating researches about aberrant lncRNAs have emerged in OS, which has been shown to contribute to OS occurrence and progression by inhibiting tumor suppressor genes or promoting oncogene expression. For example, lncRNA MALAT1 may promote OS cell growth through inhibition of miR-376A, leading to increased expression of TGFA [10]. Silencing lncRNA PVT1 by siRNA inhibited OS cell proliferation, migration and invasion and promoted apoptosis and cell cycle arrest in OS cells via miR-195 regulated BCL2, CCND1, and FASN protein expression [11]. Abnormal lncRNA Xist expression in cancer tissues, such as hepatocellular carcinoma [12], breast cancer [13], lung cancer [14] and ovarian cancer [15], suggests that Xist is significant in cancer pathogenesis and progression. However, its expression and functions in OS are still elusive and need to be investigated. The aim of this study was to identify the role of Xist in the regulation of OS pathogenesis and progression.

## RESULTS

### LncRNA Xist is overexpressed in OS and significantly correlated with tumor size

**Figure 1:**
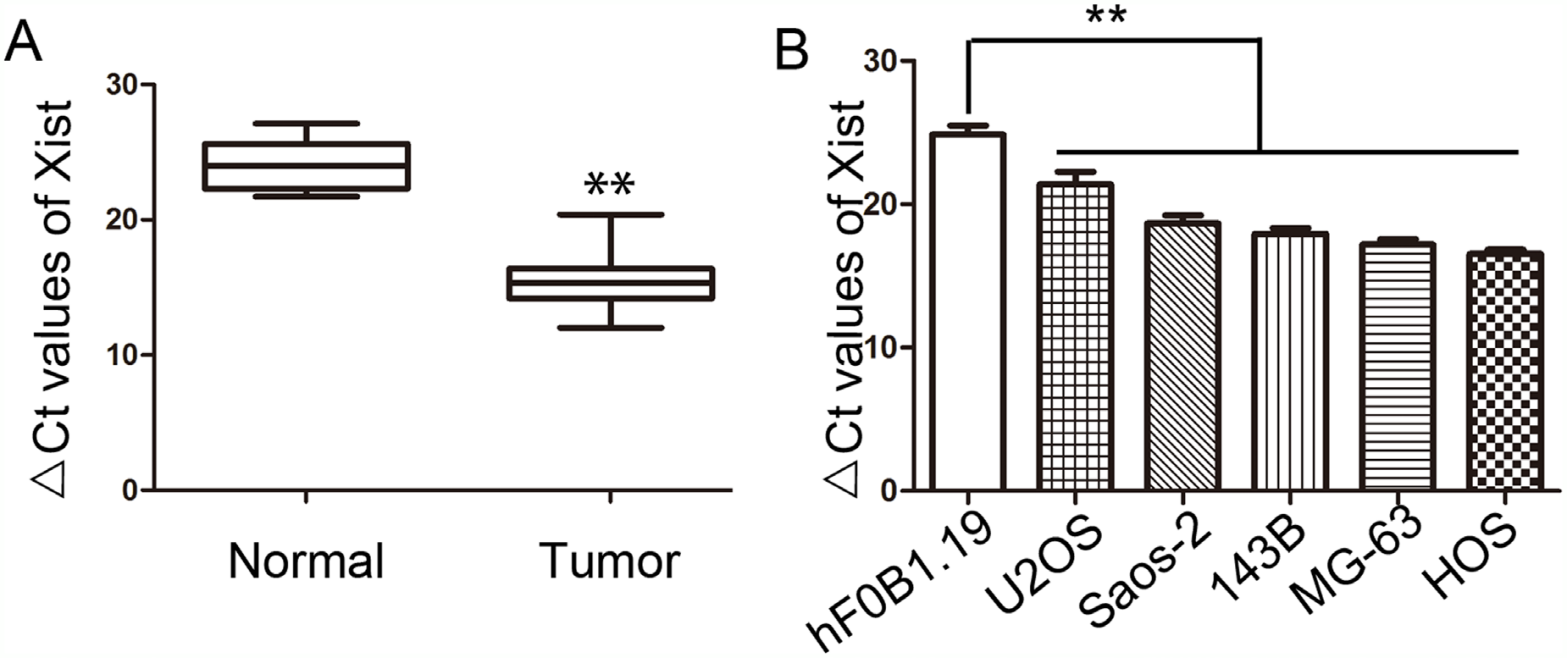
Expression of Xist in OS tissues and OS cell lines **(A)** Relative expression of Xist in OS tissues and the corresponding non-tumor tissues were detected by qRT-PCR. **(B)** Relative expression of Xist in five OS cell lines and osteoblastic cell line hFOB1.19 were detected by qRT-PCR. Data were shown using 2^-ΔCT^ values. GAPDH was used for normalization. ^**^P<0.01.

We collected OS tissues and corresponding noncancerous tissues from 66 patients. The expression level of Xist was measured by qRT-PCR and the results showed that Xist was expressed at higher levels in OS tissues compared with corresponding noncancerous tissues (Figure 1A). Xist expression was also measured in U2OS, Saos-2, 143B, MG-63 and HOS cell lines, which was obviously higher than the normal human osteoblastic cell line hFOB1.19 (Figure 1B).

Given the remarkable upregulation of Xist in OS tissues, we carried on to study the correlation between Xist expression and clinicopathologic characteristics of OS patients. We divided all patients into two groups based on its expression level: the Xist high-expression group (n = 40) and the Xist low-expression group (n = 26). The results showed that the Xist level was remarkably correlated with tumor size (*P* = 0.034) and there was no relationship between Xist expression level and age, gender, location, clinical stage or distant metastasis (Table 1).

**Table 1:** Association between Xist expression and clinicopathological factors of osteosarcoma patients

Variables	Number of cases	Xist expression		*P*
		High (n=40)	Low(n=26)	
Gender				
Female	31	19	12	0.915
Male	35	21	14	
Age(years)				
≥20	35	22	13	0.691
<20	31	18	13	
Tumor size				
≤8cm	30	14	16	
>8cm	36	26	10	0.034
Location				
Tibia/femur	41	22	19	
Elsewhere	25	18	7	0.139
Clinical stage				
I/II	28	17	15	
III	38	23	11	0.228
Distant metastasis				
Absent	40	29	21	
Present	16	11	5	0.444

### Manipulation of Xist levels in OS cells

**Figure 2:**
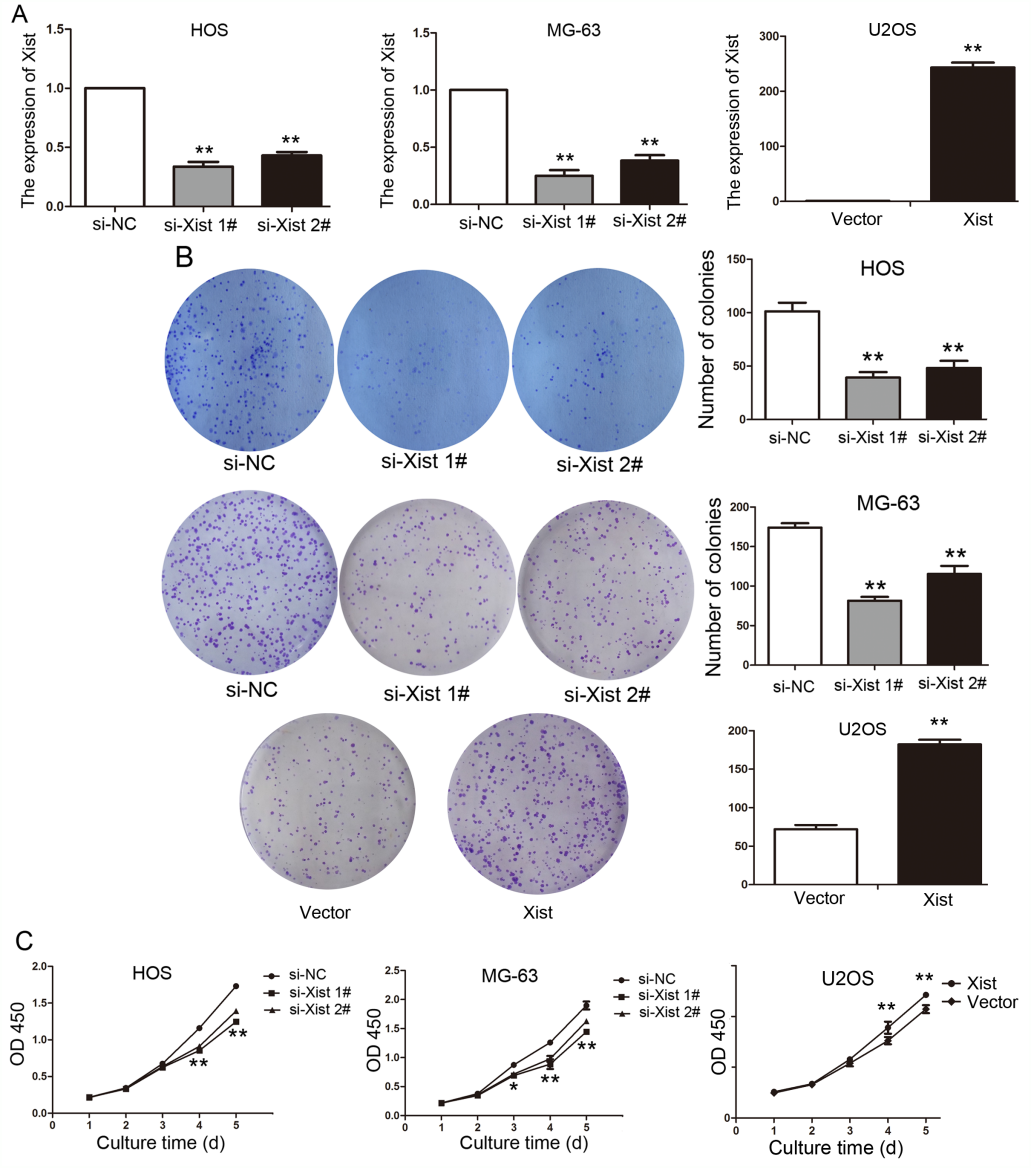
Xist regulates OS cell proliferation *in vitro* **(A)** The results of qPCR determined that the relative expression level of Xist by transfection with si-Xist or Xist vector in OS cells, compared with the transfection with control vector. **(B)** Representative photographs and quantitative analysis of plate colony formation of OS cells transfected with si-Xist, Xist vector or control vector. **(C)** CCK-8 assays indicated that decreased Xist expression inhibited HOS, MG-63 cells proliferation and increased Xist expression promoted U2OS cells proliferation. Data are represented as mean±SD of three independent experiments. ^*^P< 0.05; ^**^P < 0.01 compared with control group.

Based on the above findings, we chose HOS, MG-63 and U2OS cells. To manipulate the Xist level in OS cells, Xist siRNA was transfected into HOS and MG-63 cells, two effective interference target sequences of Xist were used, and Xist overexpression vector was transfected into U2OS cells, After transfection, we examined the Xist levels by qRT-PCR. The expression of Xist significantly reduced in HOS and MG-63 cells and significantly increased in U2OS cells (Figure 2A).

### Xist promotes OS cell proliferation

To assess the function of Xist in OS, we investigated the effect of targeted knockdown and up-regulated of Xist on cell proliferation. Colony-formation assays indicated that the colony number were decreased in si-Xist-transfected HOS and MG-63cells (*P*<0.01, Figure 2B), Similarly, the results of CCK-8 assays revealed that cell growth was markedly inhibited following knockdown of Xist in OS cells compared with control cells (*P*<0.05, Figure 2C). We observed the opposite results in U2OS cells, after Xist expression was up-regulated (*P*<0.05, Figure 2B, 2C).

### Xist is involved in OS cell cycle and apoptosis

**Figure 3:**
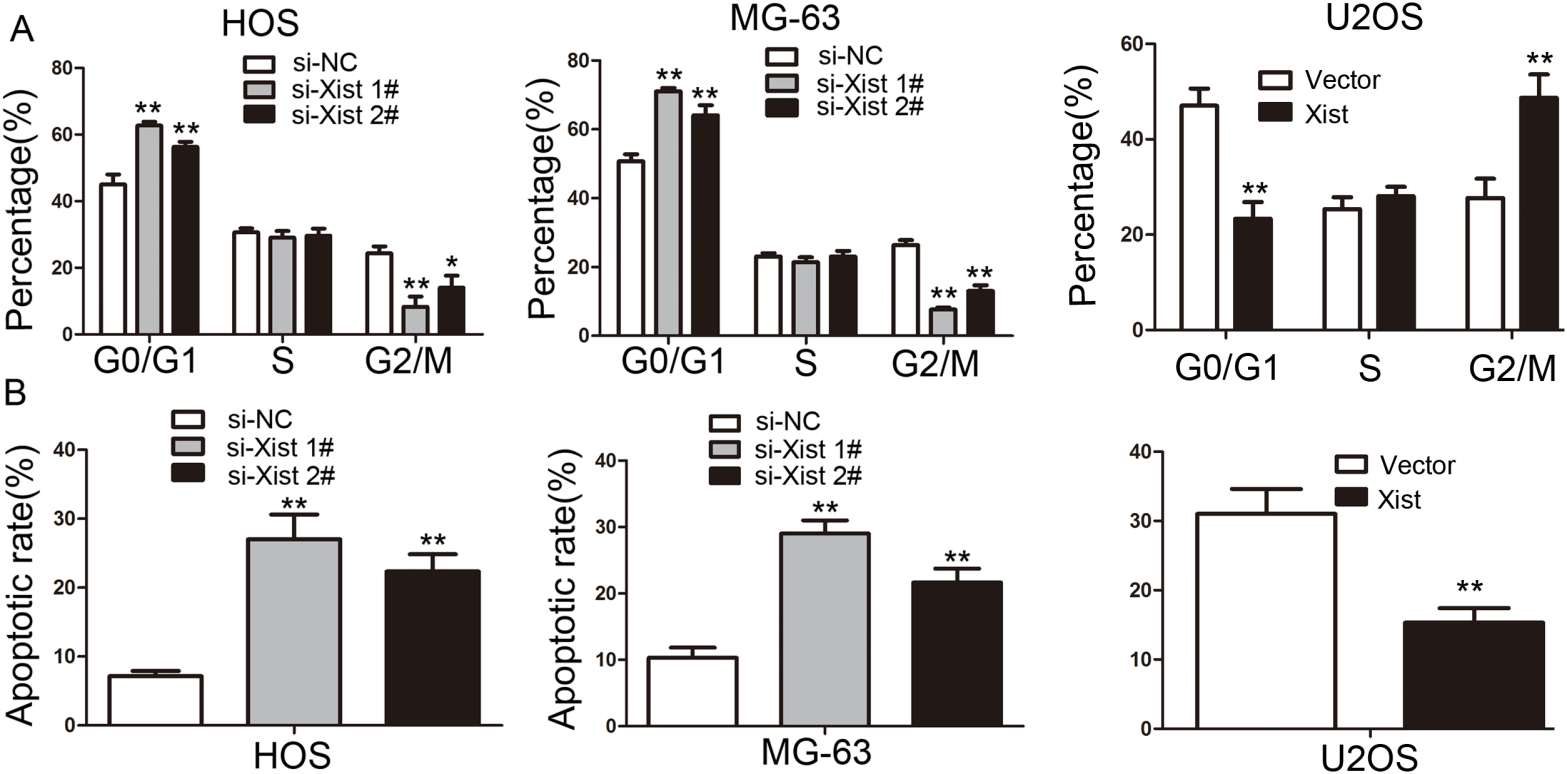
The effect of Xist on cell cycle distribution and apoptosis of OS cells **(A)** Cell cycle analysis showed that the Xist influenced the distribution of G0/G1 and G2/M phase cells. **(B)** The results of apoptosis assays showed that inhibition of the Xist promoted HOS and MG-63 cells apoptosis, and Xist overexpression inhibited U2OS cells apoptosis. Data are represented as mean±SD of three independent experiments. ^*^P< 0.05; ^**^P < 0.01 compared with control group.

Flow cytometry analysis was performed to clarify the mechanism of Xist-mediated cell growth inhibition. si-Xist in HOS and MG-63 cells were significantly increased percentage of cells in the G0/G1 phase, alone with a decrease in G2/M-phase cells (*P*<0.05, Figure 3A). Moreover, flow cytometry analysis demonstrated a significantly higher percentage of apoptosis cells for si-Xist transfected HOS and MG-63 cells, while compared with control groups (*P*<0.01, Figure 3B). The opposite results were observed in U2OS cells after transfection of the Xist vector(*P*<0.01, Figure 3). Taken together, these results indicate that Xist could affect OS cell cycle and apoptosis, thus affect OS cell proliferation.

### Xist promotes migration and invasion of OS cells

**Figure 4:**
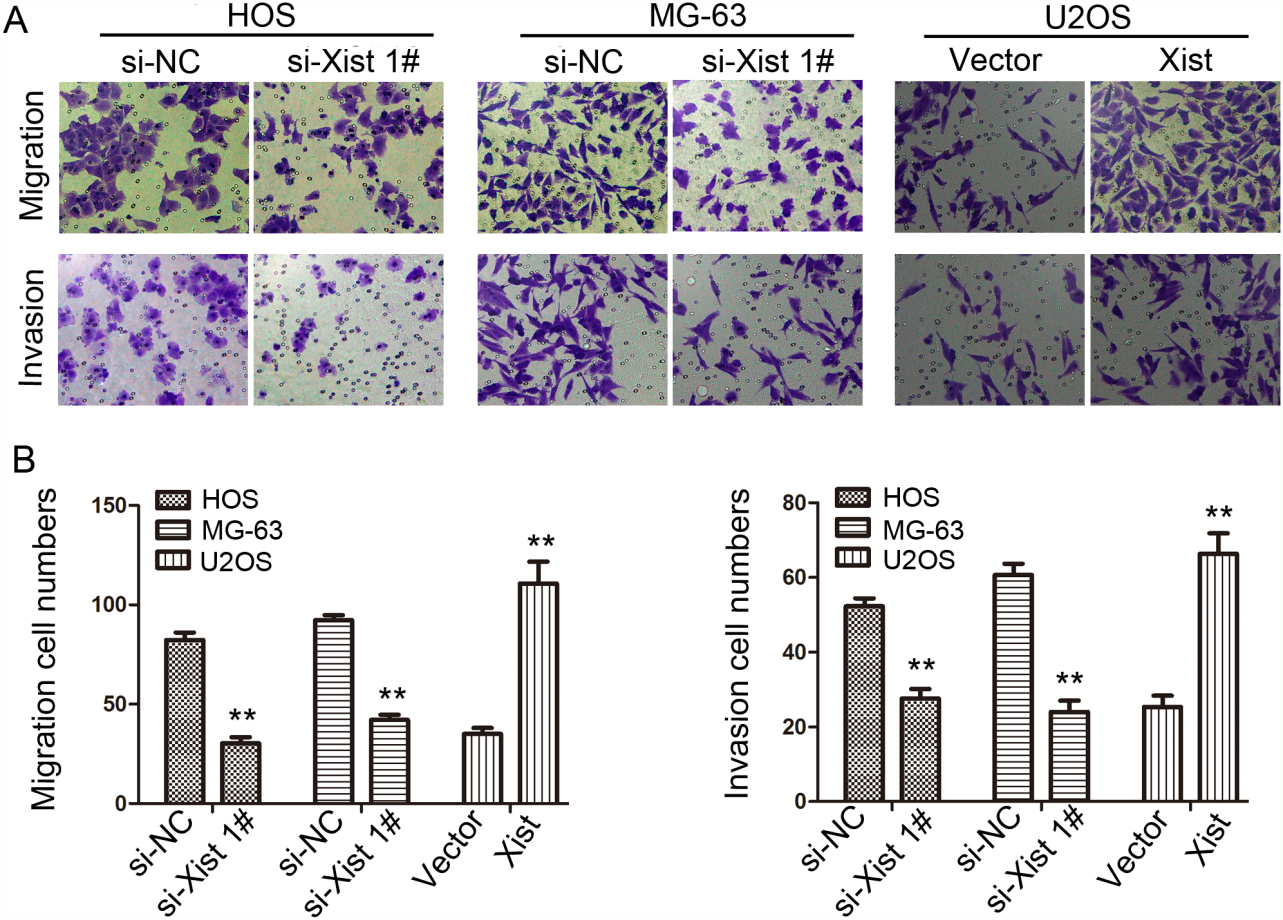
Effects of Xist on migration and invasion ability in OS cells (200×) **(A)** Transwell assay. Photographs show cells that travelled through the micropore membrane with or without Matrigel. **(B)** Histograms showed the numbers of migration cells and invasion cells. Data are represented as mean±SD of three independent experiments. ^**^P < 0.01 compared with control group.

To investigate the influence of Xsit on migration and invasion of OS cells, we next performed transwell migration and invasion assays. Downregulation of Xist suppressed migration and invasion of MG-63 and HOS cells. Xist expression in U2OS cell was upregulated and then performed to examine the accuracy of the finding. As expected, opposite results were observed in transwell migration and invasion assays (*P*<0.01, Figure 4).

### Silencing Xist inhibits tumorigenicity *in vivo*

**Figure 5:**
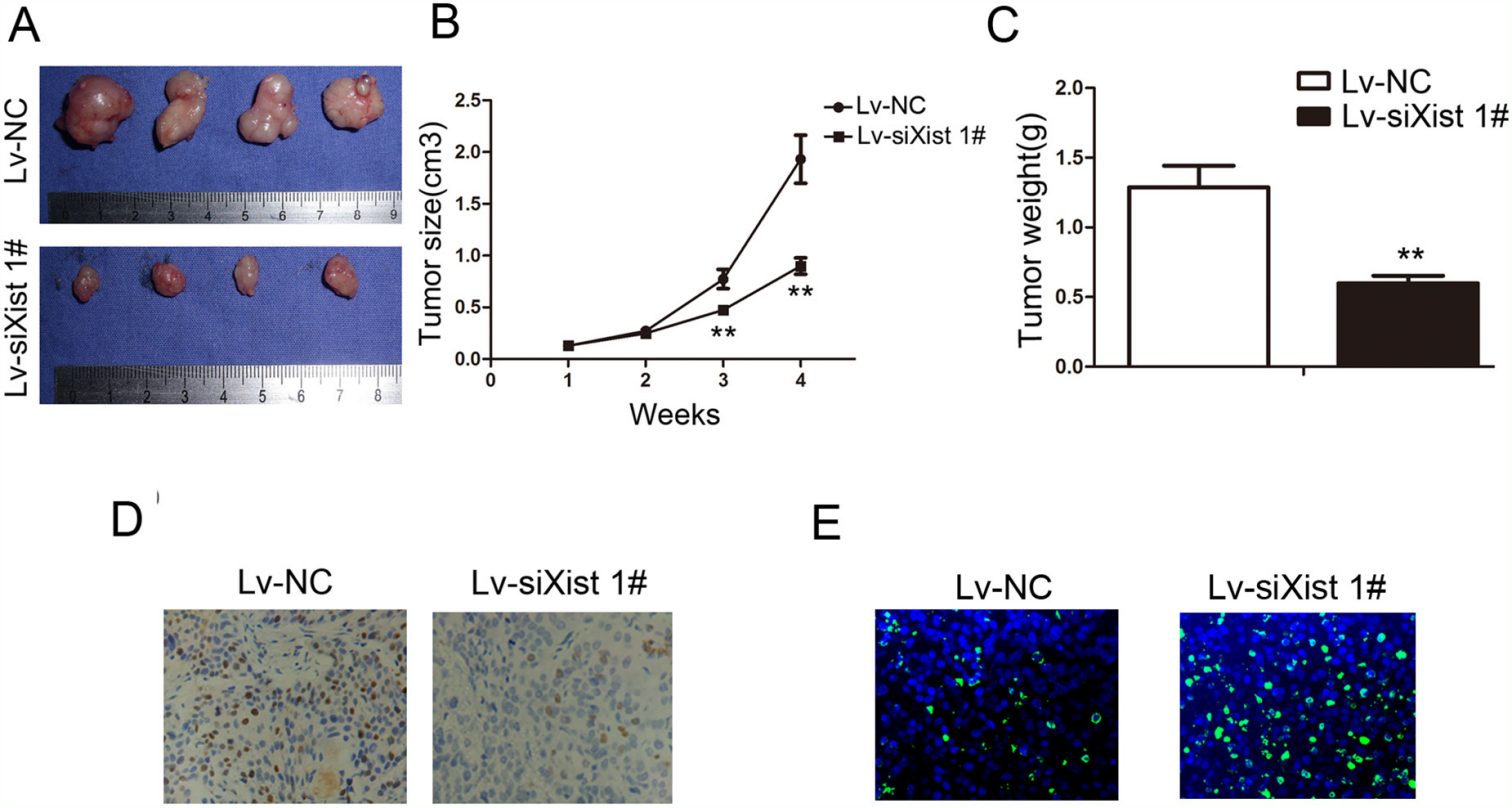
Suppressing Xist inhibits tumorigenicity and proliferation *in vivo* **(A)** Photograph of tumors harvested from LV-NC and LV-siXist 1# cells in nude mice. **(B)** The tumor volumes kinetics of tumors in nude mice. Tumor diameters were measured every week. **(C)** The tumor weights were lower in LV-siXist 1# group than that in the control group. **(D)** The tumor sections underwent IHC staining using antibodies against Ki-67. Data are represented as mean±SD of three independent experiments. ^**^P < 0.01 compared with control group. **(E)** The tumor sections underwent TUNEL staining using TUNEL apoptosis detection kit.

We have demonstrated that incRNA Xist suppresses OS cell growth *in vitro*, therefore we continued to investigate if Xist has any impact upon tumorigenicity *in vivo*. We injected Lv-siXist 1# or Lv-NC-transfected MG-63 cells into nude mice. Consistent with our *in vitro* results, the growth of MG-63 cells in Lv-siXist 1# group was significantly slower than in Lv-NC group (*P*<0.01, Figure 5A and 5B). In addition, 4 weeks after tumor cell inoculation, the weight of tumors derived from Lv-siXist 1# group was significantly less than that of control group (*P*<0.01, Figure 5C). We next examined the expression of ki-67 in transplantation tumor samples by IHC. As shown in Figure 5D, levels of ki-67 protein was decreased in the Lv-siXist 1# group compared with Lv-NC group. Consistent with this result, percentage of apoptotic cells detected by Tunel method was increased in the Lv-siXist 1# group compared with Lv-NC group (Figure 5E).

### Xist regulates epigenetic repression of P21 to influence cell cycle

**Figure 6:**
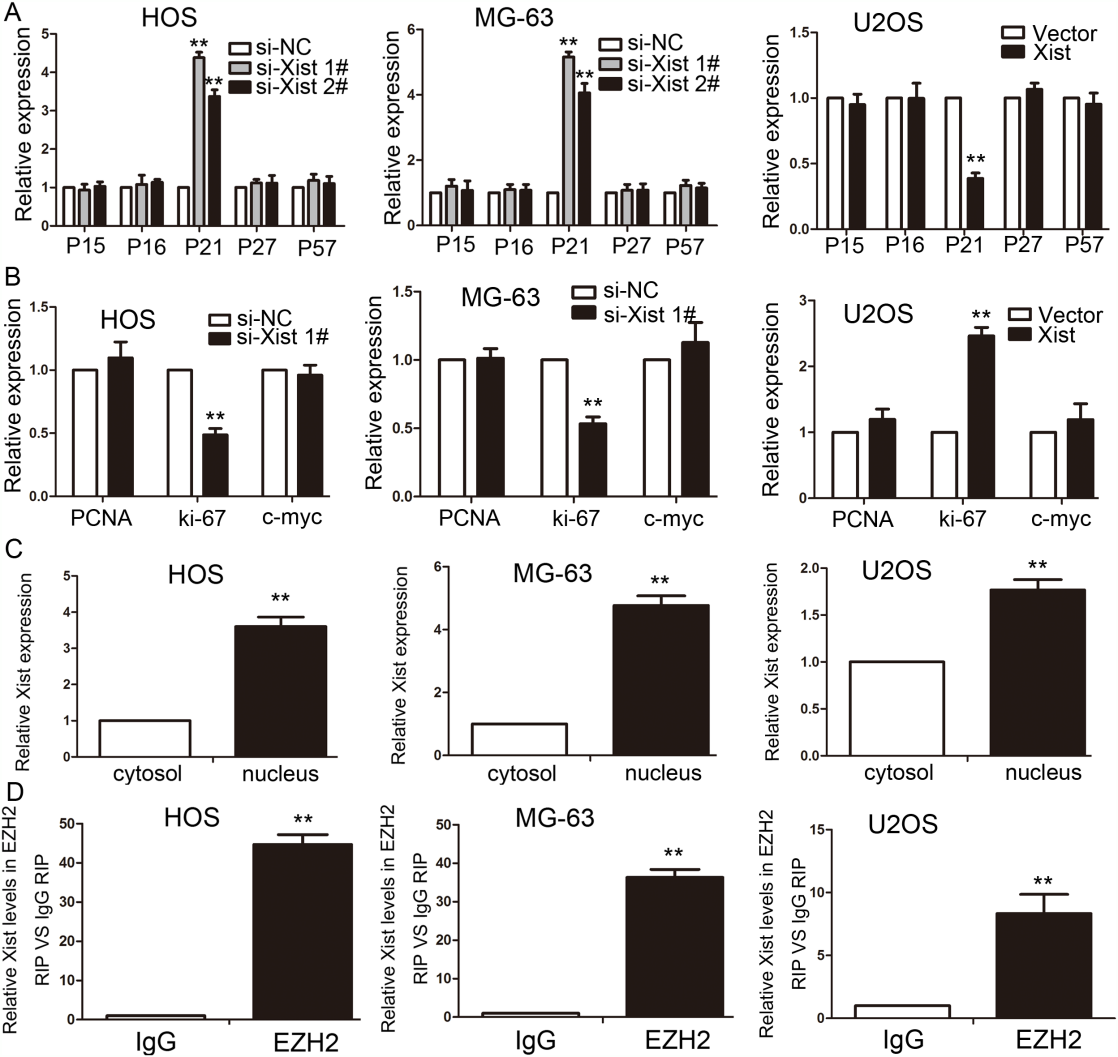
Xist is associated with EZH2 in OS **(A-B)** The expression of CKIs (A) and proliferation marker (B) were determined after knockdown or overexpress of Xist using qRT-PCR. **(C)** Xist nuclear localization, as identified using qRT-PCR in fractionated HOS, MG-63 and U2OS cells. After nuclear and cytosolic separation, RNA expression levels were measured by qRT-PCR. **(D)** Relative expression of Xist in HOS, MG-63 and U2OS cells were detected by RIP and qRT-PCR. IgG was used for normalization. Data are represented as mean±SD of three independent experiments. ^**^P < 0.01 compared with control group.

**Figure 7:**
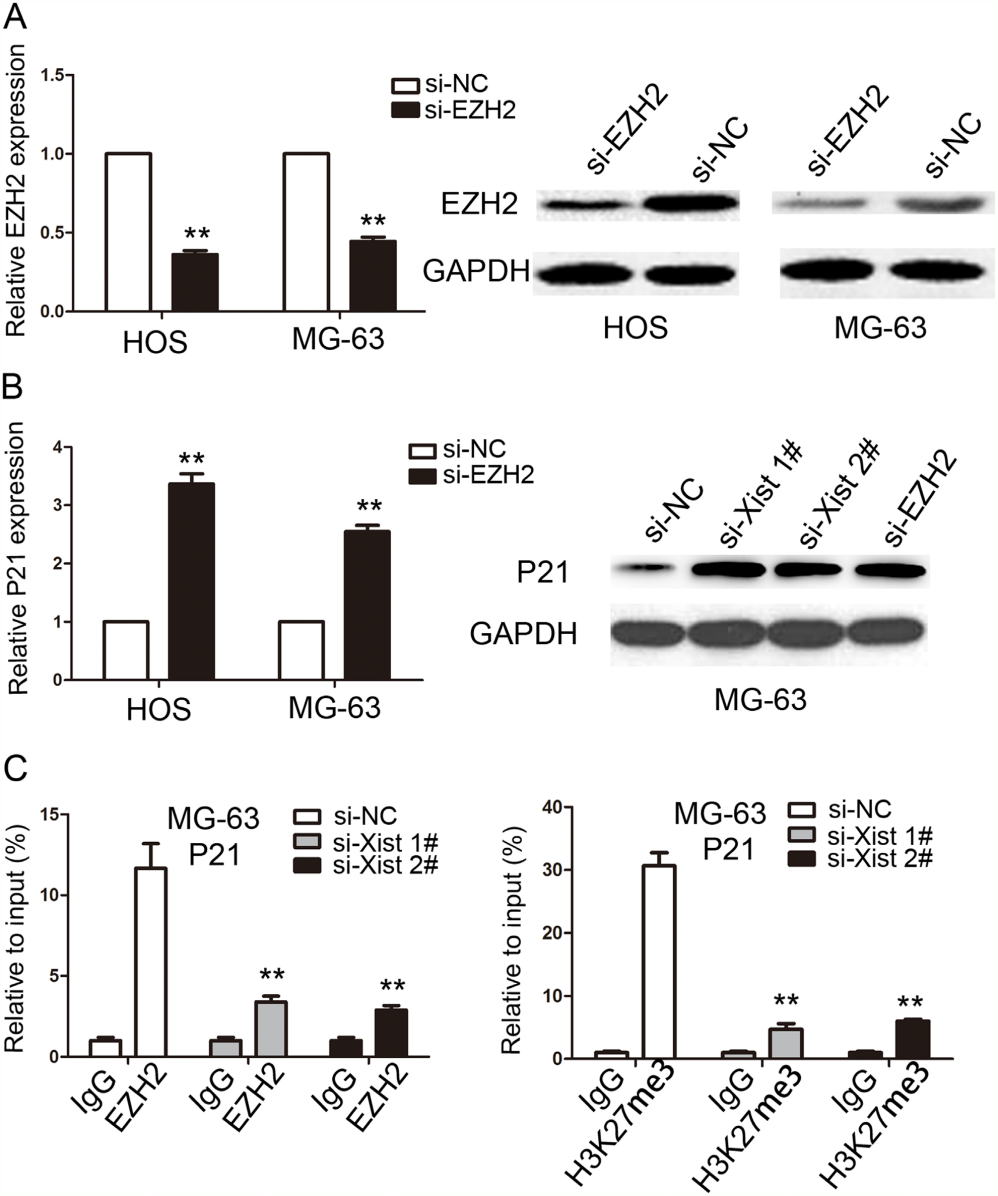
Xist epigenetically regulated the expression of P21 and thus mediated OS cell proliferation **(A)** The expression of EZH2 in HOS and MG-63 cells were determined by qRT-PCR and western blot after cells transfected with si-EZH2 or si-NC. **(B)** The expression of P21 in HOS and MG-63 cells were determined by qRT-PCR and western blot after cells transfected with si-EZH2 or si-Xist. **(C)** ChIP-qPCR of EZH2 and H3K27me3 of the promoter region of P21 after siRNA treatment targeting Xist in MG-63 cells. IgG antibody was used as a negative control. Data are represented as mean±SD of three independent experiments. ^**^P < 0.01 compared with control group.

**Figure 8:**
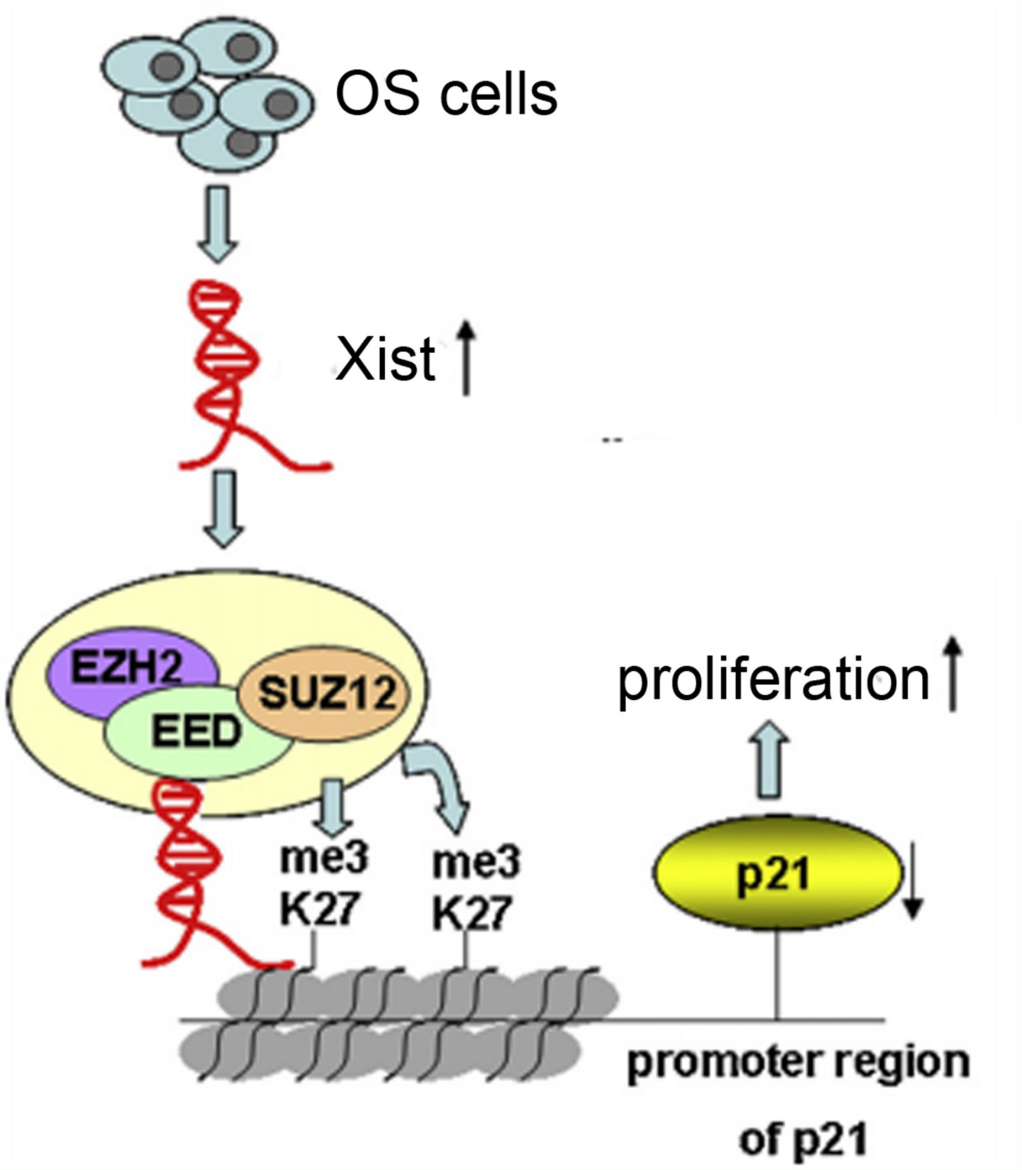
Xist, via EZH2/H3K27me3, is involved in proliferation of OS cells In OS cells, Xist interaction with thePRC2 complex, leading to enrichment of H3K27me3 in promoter region of P21, which results in cell cycle aberrations and OS cell proliferation.

The results of the study have shown that Xist has an effect on the cell proliferation and cell cycle, so we investigated the mRNA expression of proliferation marker (PCNA, ki-67 and c-myc) and CDK inhibitors by using qRT-PCR method, and the results showed that Xist could inhibit the expression of P21 and promote the expression of ki-67 (Figure 6A, 6B). We researched the expression of Xist in nucleus or cytosol by qRT-PCR to explore the mechanism of Xist in the role of OS cell cycle, the results indicated that Xist expression was significantly higher in nucleus than cytosol (Figure 6C), suggested that Xist may regulate cell cycle protein at the transcriptional level. EZH2 is a part of the polycomb repressive complex 2 (PRC2) complex, and PRC2 can bind to Xist RNA and regulates histone modification status of target genes. We used RNA immunoprecipitation (RIP) assay to research the correlation between PRC2 and Xist in OS cells, then we found that endogenous Xist was significantly enriched in the anti-EZH2 RNA-IP fraction when compared with the IgG fraction in OS cell lines (Figure 6D). We silenced the expression of EZH2 in HOS and MG-63 cells by tranfection with si-EZH2 (Figure 7A) to investigated the role of PRC2 in co-regulating the suppression of Xist-suppressed P21. The mRNA expression of P21 was upregulated after knockdown of Xist in HOS and MG-63 cells, and P21 protein expression level was also upregulated after transfection with si-Xist or si-EZH2(Figure 7B). In order to investigate whether Xist participates in transcriptional inhibition by enriching EZH2 into P21 gene promoter, we performed chromatin ChIP analysis after Xist knockdown. The ChIP results indicated that knockdown of Xist reduced the binding of EZH2 and H3K27me3 levels in the P21 promoter region (Figure 7C). These results suggest that Xist is required to target EZH2 occupancy and works to epigenetically regulate the expression of P21(Figure 8).

## DISCUSSION

LncRNAs have been identified to be involved in multiple biological functions, and also play a vital role in OS carcinogenesis [16, 17, 18]. LncRNA X-inactive specific transcript (Xist) is a product of the Xist gene, which mainly regulates the chromosome X chromosome inactivation and the Xist gene is encoded only by the inactivated X chromosome [15]. Studies have shown that Xist is highly expressed in certain tumors, such as glioblastoma [19], breast cancer [20] and ovarian cancer [21], indicating that Xist can be used as a biomarker for the diagnosis of neoplastic diseases. However, Xist expression level in OS is still unknown. In the current study, we, for the first time, show that Xist is highly expressed in OS tissues compared with adjacent non-tumor tissues and its high expression relates to tumor size. In order to clarify the mechanism of this abnormal expression, we found that Xist promotes OS cell growth ability by regulating P21, which may strengthen the understanding of the development of OS.

As a newly discovered carcinogens, several studies demonstrate that Xist, contributes tumor development mainly by promoting cancer cell proliferation and migration. For example, Xist was highly expressed in nasopharyngeal carcinoma (NPC) and promoted tumor NPC cell growth *in vitro* and *in vivo* through miR-34a-5p-E2F3 axis [22]. And also, Xist was up-regulated in gastric cancer and knockdown of Xist suppressed cell proliferation, migration and invasion through regulating lncRNA Xist/miR-101/EZH2 axis [23]. Similar to these findings, we found that knockdown of Xist inhibits OS cell proliferation, colony forming ability, and also induces G0/G1 cell cycle arrest and promotes cell apoptosis. These resultes may justify Xist high expression in OS and clinical association we discovered.

PRC2 is one important complexes of polycomb group genes (PcG) complexes, which mainly includes enhancer of zeste homolog 2 (EZH2), suppressor of zeste 12 homologue (SUZ12), embryonic ectoderm development (EED) and Yin Yang 1(YYl), and it plays its gene silencing function mainly by histone modification. EZH2 is the only subunit with enzyme activity in the PRC2 complex, but SUZ12, EED and YYl are indispensable components for PRC2 enzymatic activity [24]. As an important epigenetic transcriptional inhibitor, PRC2 complex plays an important role in embryonic development, stem cell maintenance, X chromosome inactivation, tumor occurrence and metastasis [25]. EZH2, as a member of PRC2 complex, plays a role in the regulation of gene expression by stimulating histone methyltransferase activation at the 27th lysine of histone H3. When the PRC2 complex is targeted at the target gene, its own interaction between the sub-proteins affects gene regulation, lncRNA is also involved in regulating the aggregation of PRC2 and influencing the biological activity and function of PRC2 complex, which is indirectly involved in the regulation of gene expression [26]. IncRNA UBC1 can physically associates with PRC2 complex and regulates histone modification status of target genes in bladder cancer [27]. In colorectal cancer, incRNA BLACAT1 can repress p15 expression by binding to PRC2 (EZH2 part), thus contributing to the regulation of CRC cell cycle and proliferation [28]. In this research, we illustrated binding relationship between Xist and EZH2 in OS cells, and we further confirmed that Xist could mediate epigenetic regulation of P21through binding with EZH2. These resultes gives us a new perspective on the role of Xist in OS.

In summary, our data demonstrated that Xist is up-regulated in OS tissues and OS cell lines, and associated with tumor size in OS patients. Knockdown of Xist repressed cell proliferation, induced G0/G1 cell cycle arrest and promoted cell apoptosis. In addition, Xist exercised its oncogenic role is partially through its epigenetically silencing of the P21 expression through binding with EZH2 (a member of PRC2). Our findings provide new insights into the function of lncRNAs in the development of OS and suggest that Xist represents a potential therapeutic target for the treatment of OS.

## MATERIALS AND METHODS

### Human tissue specimens and cell culture

Fresh-frozen OS tissues and paired adjacent non-tumor tissues were obtained from 66 patients who undergoing surgery at the Shanghai Tenth People's Hospital from 2012 to 2016. The study was approved by the ethics committee of the Ethical Committee of Shanghai Tenth People's Hospital, Tong Ji University School of Medicine and informed consent was obtained from all patients. All the patients did not receive any treatment before the operation. All the specimens were immediately frozen in tubes containing RNA-later preservation liquid after removal and stored at liquid nitrogen until RNA extraction. Histological typing was performed by at least two expert pathologists, working independently in a double-blinded fashion.

Human OS cell lines (U2OS, Saos-2, 143B, MG-63, and HOS) and the normal human osteoblast cell line hFOB 1.19 cells were obtained from either the American Type Culture Collection or the type Culture Collection of Chinese Academy of Sciences (Shanghai, China). These cells were cultured were cultured in RPMI 1640 containing 10% calf serum, streptomycin (100 IU/ml) and penicillin (100 IU/ml) in a 5% CO_2_ atmosphere at 37°C.

### RNA extraction and real-time PCR analysis

Total RNA was extracted from cells and tissue samples using trizol reagent (Invitrogen, Carlsbad, CA, USA) according to the manufacturer's protocol. qRT-PCR assays were performed to detect lncRNA Xist and EZH2 expression using the reverse transcription kit (Toyobo, Osaka, Japan) and SYBR Green reagent (Applied Biosystems, Foster City, CA, USA) according to the manufacturer's instructions. GAPDH was used as the endogenous control. The up-regulation of Xist was considered to be positive only when the Xist expression score was greater than 1.7, as described elsewhere [29]. The PCR primers designed for Xist: 5'-GCATAACTCGGCTTAGGGCT-3'(forward) and 5'- TCCTCTGCCTGACCTGCTAT -3'(reverse); for EZH2: 5'- TGCAGTTGCTTCAGTACCCATAAT -3'(forward) and 5'- ATCCCCGTGTACTTTCCCATCATAAT -3'(reverse); for GAPDH: 5'- GTAGCCCAGGATGCCCTTGA -30 (forward) and 5'- GGACCTGACCTGCCGTCTAG -30(reverse). The real-time PCRs were performed in triplicate.

### Cell transfection

Interfering lentivirus, small interfering RNA (siRNA), overexpression vector and negative control vector were purchased from GenePharma (Shanghai, China). Briefly, lentiviral small hairpin RNA (shRNA) targeting Xist was designed and cloned into the pLV-H1TetO-GFP-Puro vector according to manufacturer's instructions (Biosettia Inc., San Diego, CA, USA). The viruses were packaged in 293T cells according to standard protocols, and the virus particles were harvested 72 h later. MG-63 cells were infected with virus particles plus 8 μg/mL Polybrene. The target sequences for Xist siRNAs were as follows: si-Xist 1#: GUAUCCUAUUUGCACGCUAtt, si-Xist 2#: GCCCUUCUCUUCGAACUGUtt. siRNA or si-NC: GAGTCTCCGTTGGTTGTTCTCGCTA were transfected into HOS and MG-63 cells. HOS and MG-63 cells were grown on six-well plates to confluency and transfected using Lipofectamine 2000 Reagent (Invitrogen) according to the manufacturer's instructions. Forty-eight hours after transfection, transfection efficiency was calculated by qRT-PCR or western blot.

### Protein extraction and western blot analysis

Cells were lysed using RIPA protein extraction buffer supplemented with a protease inhibitor cocktail (Pierce, Rockford, IL, USA), and protein concentration was measured using the BCA Protein Assay Kit (Pierce). 50 μg protein was separated by 10% SDS-polyacrylamide gel electrophoresis (SDS-PAGE), then transferred to a polyvinylidene fluoride (PVDF) membrane and incubated with specific antibodies. Antibodies for P21, EZH2 and GAPDH were purchased from Abcam (Cambridge, MA, USA), used in a dilution of 1:1000. GAPDH was used as a control.

### Cell proliferation analysis

Cell proliferation ability was determined by performing the colony formation assay and CCK-8 assay. For the colony formation assay, 1000 cells were placed in a six-well plate and cultured with RPMI 1640 medium containing 10 % FBS for 10 days. The number of colonies was counted using a phase-contrast microscope after staining with 0.5% crystal violet solution. For CCK-8 assay, 1000 cells were seeded into a 96-well plate with 200μL RPMI 1640 and cultured at 37 °C. Each well was added with 20 μl CCK-8 solution. After 2h incubatation, absorbance was measured at 450 nm with a Microplate Autoreader (Bio-Rad, Hercules, CA, USA). All experiments were performed in triplicate.

### Flow cytometric analysis of cell cycle and apoptosis

The HOS and MG-63 cells, transiently transfected with si-Xist or si-NC, were collected 48h after transfection by trypsinization. For cell cycle analysis, OS cells were first fixed using 70% ethanol and stored at 4 °C overnight. Followed by the next day, the OS cells were washed with PBS, treated with 50 mg/ml RNase A and stained with 50 mg/ml propidium iodide (PI) for 30 min in the dark. Cell cycle distribution were analyzed by flow cytometry (FACSCalibur, Becton-Dickinson). For apoptosis assay, cells were harvested and were resuspended in 1× binding buffer. After double staining with Annexin V and PI (BD Pharmingen, USA), cells were analyzed according to manufacturer's instructions to detect early and late apoptosis of cells. All the samples were assayed in triplicate.

### Transwell assay

For the migration assay, approximately 1.0 × 10^5^ of cells in 100 μL of serumfree medium were placed in the upper chamber (Corning Costar, NY, USA), which was not coated with Matrigel, whereas 600 μL of the same medium with 10% FBS was placed in the lower chamber. After 24 h, the cells that had migrated were stained with 0.5% crystal violet solution for 15 min, and counted under a microscope using five random fields (magnification: 200×). For the invasion assay, a procedure described in the cell migration assay was performed, except that the upper chamber was pre-coated with Matrigel (BD Bioscience, CA, USA). All the experiments were performed in triplicate.

### Subcellular fractionation location

PARIS Kit (Life Technologies, Carlsbad, CA, USA) was used to separate the fractions in cytosolic or nuclear according to the manufacturer's instructions.

### Chromatin immunoprecipitation (CHIP) assay

ChIP assays were performed using EZ-CHIP KIT (Millipore, Billerica, MA, USA) following the manufacturer's protocol. Antibodies against EZH2 (Abcam, CA, USA) and H3K27me3 (Abcam, CA, USA) or control IgG antibody were used to precipitate protein/DNA complex. The PCR primers designed for CHIP: 5'- GGTGTCTAGGTGCTCCAGGT -3'(forward) and 5'- GCACTCTCCAGGAGGACACA -3'(reverse). Quantification of IP DNA was performed using qPCR with SYBR Green. The CHIP assay were performed in triplicate.

### RNA immunoprecipitation (RIP) assay

Magna RIP RNA-Binding Protein Immunoprecipitation Kit (Millipore, Billerica, MA, USA) was used to perform RIP experiments according to the manufacturer's instructions. Antibodies for RIP assays against EZH2 were purchased from Abcam and IgG was used as negative control. The PCR primers designed for RIP : 5'- AGCTCCTCGGACAGCTGTAA -3'(forward) and 5'- CTCCAGATAGCTGGCAACC -3'(reverse). The RIP assay were performed in triplicate.

### Xenograft model

Animal studies in this research completly complied with protocols approved by the Committee on Animal Care in Tong Ji University School of Medicine. Four-week-old male BALB/C nude mice were purchased from the Institute of Zoology, Chinese Academy of Sciences of Shanghai. All experiments were performed A number of 1×10^6^ Lv-siXist 1# or Lv-NC cells were subcutaneously inoculated into the flanks of mice and each group contained five nude mice. The tumor volumes (length × width^2^ × 0.5) were measured every weeks in mice from the Lv-siXist 1# or Lv-NC groups. Four weeks after injection, the mice were sacrificed and the harvested tumors were weighed and examined Ki-67 staining and TUNEL staining.

### Statistical analysis

The Spearman test, Student's t test, and one-way ANOVA were performed to analyze the data with the SPSS 15.0 software (SPSS, Chicago, IL, USA). All data are expressed as the means ± SD. *P* < 0.05 was considered significant, *P* < 0.01 was considered highly significant.
